# Microarray data of transcriptome shifts in blood cell subsets during S1P receptor modulator therapy

**DOI:** 10.1038/sdata.2018.145

**Published:** 2018-07-24

**Authors:** Dirk Koczan, Brit Fitzner, Uwe Klaus Zettl, Michael Hecker

**Affiliations:** 1 University of Rostock, Institute of Immunology, Schillingallee 70, 18057 Rostock, Germany; 2 Steinbeis Transfer Centre for Proteome Analysis, Schillingallee 70, 18057 Rostock, Germany; 3 University of Rostock, Department of Neurology, Division of Neuroimmunology, Gehlsheimer Str. 20, 18147 Rostock, Germany

**Keywords:** Neuroimmunology, Multiple sclerosis

## Abstract

Treatment with fingolimod, a sphingosine-1-phosphate (S1P) receptor modulator, prevents the egress of immune cell subpopulations from lymphoid tissues into the blood. We obtained peripheral blood samples from patients with relapsing multiple sclerosis before the initiation of fingolimod therapy, after one day and after 3 months. To investigate the differential expression induced by the drug, five different cell populations were isolated. We then employed 150 Human Transcriptome Arrays (HTA 2.0) interrogating >245,000 protein-coding and >40,000 non-coding transcript isoforms. After 3 months of treatment, CD4+ and CD8+ T-cells showed huge transcriptome shifts, whereas the profiles of B-cells (CD19+) were slightly altered and those of monocytes (CD14+) and natural killer cells (CD56+) remained unaffected. With >6 million probes for exons and splice junctions, our large HTA 2.0 dataset provides a deep view into alternative splicing patterns in immune cell subsets. Our data may also be useful for comparing the effects on gene expression signatures of novel S1P receptor modulators, which are currently tested in clinical trials for other autoimmune and neurodegenerative diseases.

## Background & Summary

Multiple sclerosis (MS) is an immune-mediated disease of the brain and spinal cord, which is characterised by the infiltration of autoaggressive lymphocytes through the blood-brain barrier^1,2^. Approximately 85% of the 2.3 million people suffering from MS worldwide have a relapsing-remitting course of the disease (RRMS), which is distinguished from the progressive course^3^. Several immunomodulatory therapies for MS are applied by injection or intravenous infusion^4^. Fingolimod has been the first of a new class of oral drugs approved for RRMS^5^. It acts as a sphingosine-1-phosphate (S1P) receptor modulator as a structural analogue of natural S1P^6^. Sphingosine is in its phosphorylated form important for the cellular trafficking between the peripheral blood and lymph nodes. The S1P gradient is triggering the egress of recirculating cells, which are expressing S1P receptors, by overwriting the retention signals mediated by lymph node homing receptors like CCR7^7^. However, whereas binding of S1P to its receptors is followed by internalisation and recycling, binding of phosphorylated fingolimod causes long-lasting internalisation and degradation of the receptors^6^. In consequence, the egress of certain cell populations from lymphoid tissues is inhibited, which changes the relative frequencies of circulating lymphocytes. This leads to less autoreactive T- and B-cells in the peripheral blood and reduced migration of such cells across the blood-brain barrier. CCR7+ naïve T-cells and CCR7+ central memory T-cells are strongly trapped in lymph nodes in response to fingolimod, whereas CCR7- effector memory T-cells are spared. After 3 months of therapy, the overall proportion of CD4+ T-cells within blood lymphocytes is reduced from 46 to 6% and those of CD8+ T-cells from 20 to 15% (ref. 8). The population of CD19+ B-cells also decreases from 11 to 6% of circulating lymphocytes^8^. As a consequence, the frequencies of monocytes (CD14+) and natural killer cells (CD56+) are relatively increased within the peripheral blood mononuclear cell pool^9,10^. Despite these insights from various flow cytometry studies, there has been a lack of data on the effects of fingolimod on the transcriptome.

**Figure 1:**
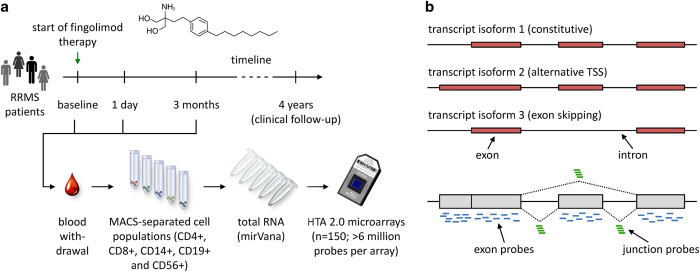
Study workflow and probe design of the transcriptome microarray. (**a**) The main steps of data acquisition are presented. Peripheral blood was obtained longitudinally at 3 different time points: before the first dose of fingolimod, after ~24 h (before the second dose) and after 3 months of therapy. Five blood cell subsets were then each enriched by magnetic-activated cell sorting (MACS) from the blood samples of 10 patients, which were diagnosed with relapsing-remitting multiple sclerosis (RRMS). Finally, total RNA was isolated and used for the high-resolution gene expression analysis with 150 Affymetrix Human Transcriptome Arrays (HTA 2.0). (**b**) Illustration of the deep coverage of the transcriptome by HTA 2.0 microarrays (adapted from Xu *et al.*^11^). Shown is the exon-intron structure of a gene encoding 3 different transcript isoforms, which result from usage of an alternative transcription start site (TSS) and exon skipping during splicing. The accurate measurement of transcript abundance is realised by about 10 oligonucleotide probes (25mers) for each exon fragment and 4 probes for each exon-exon junction. In this case, the probe signal intensities can be summarised to either 7 exon-level probe sets or 1 gene-level probe set comprising all 52 probes mapping to the gene.

The goal of our study was to monitor the differential gene expression in 5 cell populations, which were defined by the cell surface markers CD4, CD8, CD14, CD19 and CD56, in response to fingolimod treatment. In total, 150 high density Human Transcriptome Arrays (HTA 2.0) with more than 6 million distinct 25mer oligonucleotide probes were used for the profiling analysis of whole RNA prepared from blood samples of 10 RRMS patients at 3 different time points (before as well as ~24 h and 3 months after the start of fingolimod therapy) for each of the 5 cell populations. The signal intensities of HTA 2.0 probes can be summarised to either 70 523 gene-level probe sets, including 22,829 probe sets for non-protein-coding genes, or 914 585 exon-level probe sets. As successor of the Glue Grant human array, this array has been designed based on the GRCh37 human reference genome assembly, and it realises a new concept for the analysis of splicing isoforms^11^. Each unique exon fragment is in general interrogated by 10 oligonucleotide probes, and more than 339 000 probe sets, each designed with 4 different probes, cover exon-exon junctions (Fig. 1). This enables the transcriptome-wide exploration of alternative splicing events^12^.

The largest shift in the gene expression profile in response to the therapy with fingolimod occurred in the CD4+ cell population, followed by the CD8+ cells, and a moderate change in expression was observed for the CD19+ B-cells. This correlated with a marked decrease in the number of circulating T- and B-cells. In contrast, monocytes (CD14+) and natural killer cells (CD56+) were stable in their gene-level signatures^13–15^. When comparing the levels at the 3-month time point with the pretreatment levels using a *t*-test *p*-value of <0.001 and a fold-change of >1.5 as cut-offs, 6,489 differentially expressed genes were identified for the CD4+ cells, 861 for the CD8 cells and 42 for the B-cells (CD19+). The expression change in the CD4+ and CD8+ cells after continued daily drug administration could be mainly attributed to the fingolimod-induced lymph node homing of CCR7+ naïve T-cells and central memory T-cells. Accordingly, CCR7 mRNA expression was reduced by >50%, reflecting that CCR7- effector memory T-cells are remaining in the peripheral blood. Apart from that, direct effects on intracellular gene regulatory programs controlled by S1P pathway signaling could not be clearly evidenced in our data^13,14^. Further research might reveal to which extent the shift in the cell subsets at the transcriptional level is similar for other S1P receptor-modulating agents^16^. Moreover, our data can serve as a resource for examining alternatively spliced transcript isoforms across immune cell types.

## Methods

These methods are expanded versions of descriptions in our related work^13–15^.

### Study population

For the transcriptome analysis of each of the 5 cell subpopulations, peripheral blood samples of 10 patients of Caucasian descent, aged from 26 to 46 years, were used. The patients had a confirmed diagnosis of RRMS according to the revised McDonald criteria^17^, with disease duration ranging between 1 and 20 years. Functional disability was rated using the Expanded Disability Status Scale (EDSS)^18^. The individual baseline EDSS scores ranged from 1.5 to 5.5. All patients were previously treated with either glatiramer acetate or interferon-beta, and their therapies were switched to fingolimod (standard dose of 0.5 mg orally once a day) because of continued relapse activity. Routine medical care was applied to all patients according to the recommendations and guidelines of the German Society of Neurology. The study was approved by the local ethics committee of the University of Rostock and followed the ethical principles of the Declaration of Helsinki. All patients gave prior written informed consent to participate. The clinico-demographic data of the patients are available together with the microarray data in the Gene Expression Omnibus (GEO) database (Data Citation 1). We have updated the GEO records in May 2018 to provide 4-year clinical follow-up information of the patients.

### Sampling and cell separation

The blood samples were taken in the routine setting before the start of fingolimod therapy (baseline), the next day before the second dose (24-hour time point) as well as at a follow-up visit after the first 3 months of treatment. Ethylenediaminetetraacetic acid (EDTA) was used as anticoagulant. From each sample, 5 vials with 4 ml EDTA blood were prepared for the magnetic cell sorting. Each vial was mixed with 200 μl Whole Blood MicroBeads that were conjugated to antibodies against the cell surface markers CD4 (order no. 130-090-877), CD8 (130-090-878), CD14 (130-090-879), CD19 (130-090-880) and CD56 (130-090-875), respectively (Miltenyi Biotec, Bergisch Gladbach, Germany). The samples were then incubated for 15 min at 4 °C. The cell separation was done using a Miltenyi Biotec autoMACS Pro Separator according to the manufacturer’s instructions. By this means, magnetically labelled cells were eluted from the columns as positively selected cell fractions. A volume of 30 μl was kept for quality control by flow cytometry and another 30 μl aliquot was used for determining the absolute cell count under a conventional microscope. Eventually, 150 samples (5 cell populations×3 time points×10 patients) were stored at −20 °C until they were further processed for the gene expression analysis.

### RNA extraction and quality control

The extraction of total RNA was done with the mirVana miRNA Isolation Kit (Thermo Fisher Scientific, Waltham, MA, USA). After centrifugation, the total RNA extraction protocol started with adding a chaotropic denaturing solution disrupting the cell samples immediately, followed by an acidic phenol:chloroform extraction. Ethanol was then added to the samples, and the solution was passed through a filter cartridge containing a glass fibre filter, which bound the RNA. After 3 washing steps and a drying centrifugation step (1 min), the total RNA was recovered by elution with pre-heated (95 °C) nuclease-free water. Finally, all isolated RNA samples were quantified with a NanoDrop 1000 spectrophotometer (Thermo Fisher Scientific, Waltham, MA, USA).

### Microarray hybridisation

The gene expression profiling was performed using 200 ng (for CD8+ and CD14+ cells) or 70 ng (for CD4+, CD19+ and CD56+ cells) of total RNA. The lower amount of starting material was chosen if the minimum RNA yield across the longitudinal series of samples was relatively low. The so-called Whole-Transcript (WT) Sense Target Labelling protocol (Affymetrix) was started by introducing T7 promoter tags to all RNA molecules using (N)_6_ 3’ ends for DNA strand synthesis. After RNA strand replacement according to Eberwine *et al.*^19^, non-labelled antisense RNA (cRNA) was produced by *in vitro* transcription overnight. To avoid a 3’ bias, all RNA molecules were linearly amplified, followed by a purification step. Using the cRNA as template in a second reverse transcription reaction, single-stranded sense strand DNA was produced by adding random primers and dNTPs. Additionally, dUTP was intermixed and incorporated in the DNA. After removing the cRNA by RNaseH digestion, an endpoint fragmentation was performed by breaking the DNA strand at the unnatural dUTP residues using uracil DNA glycosylase and apurinic/apyrimidinic endonuclease 1. Desoxynucleotidyl-transferase was used to transfer the DNA labelling reagent (Biotin-11-dXTP) to the 3’ ends of the single-stranded DNA fragments. The hybridisation on HTA 2.0 microarrays (Affymetrix, Santa Clara, CA, USA) was then carried out at 45 °C in a GeneChip Hybridization Oven 645 (Affymetrix) overnight (16 h). After washing and staining in the Affymetrix Fluidics Station 450, the microarrays were scanned using the GeneChip Scanner 3000 7G (Affymetrix) at 0.7 μm resolution.

### Data processing and filtering

The Affymetrix GeneChip Command Console (AGCC) version 4.0 was used to visually inspect the scanned array images and to generate CEL files with processed pixel intensity values for each probe on the HTA 2.0 microarrays. The CEL files (n=150, 10 GB of data) were then imported to the Expression Console 1.3.1 software (Affymetrix) for probe set summarisation with the robust multi-array average (RMA) algorithm^20^ according to both the gene-level and the exon-level analysis workflow. This step included a background adjustment, a quantile normalisation and a log2 transformation of the data. The microarray data of each of the 5 blood cell subsets were processed separately. The resulting signal intensities for 70,523 probe sets (gene-level analysis) and 914,585 probe sets (exon-level analysis), respectively, were stored in CHP files (n=300, 6 GB of data). The raw data (CEL files) and the processed data (CHP files) can be found in the GEO database (Data Citation 1).

We then used the gene-level CHP files for exploring the transcriptome shifts induced by fingolimod therapy. Differentially expressed genes were determined using the Transcriptome Analysis Console (TAC) version 1.0 (Affymetrix). For each of the 5 cell populations, we always compared the pretreatment levels (before the first dose of fingolimod) with the levels at the 24-hour time point (before the second dose) and with the levels at the 3-month time point, ending up with 10 comparisons in total. Student’s two-tailed two-sample *t*-test *p*-values <0.001 and fold-changes >1.5 (on linear scale) were applied to filter the data. Additionally, false discovery rate (FDR) calculations to correct for multiple testing were done according to Benjamini and Hochberg^21^.

The highest number of differentially expressed genes was found after 3 months of fingolimod therapy for the CD4+ cell population (2,574 up/3,915 down), followed by the CD8+ cells (690 up/171 down) and the CD19+ B-cells (41 up/1 down). CD14+ monocytes (1 up/0 down) and CD56+ natural killer cells (0 up/0 down), however, were stable in expression. There was basically no expression shift in any of the five cell populations already after the first dose of fingolimod. Just the CD4+ cells showed one upregulated transcript after ~24 h compared with baseline following the filtering criteria. About one-third of the identified genes were annotated as non-coding genes, which are worth to be further investigated in the context of lymphocyte subpopulations and S1P receptor modulator treatments. For more details on this analysis of the data, the reader is referred to our previous publications^13–15^.

## Data Records

The complete transcriptome dataset has been deposited in the GEO database (Data Citation 1). Both the raw data (CEL files) and the RMA-processed data (CHP files) have been made publicly available together with detailed information on the patients with regard to demographic characteristics, individual disease activity and progression of disability during long-term clinical follow-up. As HTA 2.0 microarray data can be processed in two different ways, the data were linked to the GEO platform identifiers GPL17585 (exon-level analysis) and GPL17586 (gene-level analysis). The data of all 150 samples were further grouped in GEO SubSeries. For each of the 5 blood cell subsets, there are groups of 30 microarrays comprising the data of 10 RRMS patients at 3 study time points as specified in Table 1. Additionally, the ISA-Tab file provided with this publication includes machine-readable metadata for the study, listing the samples and subjects employed, the experimental and analytical protocols performed and the data outputs obtained.

## Technical Validation

### Preparation of total RNA

**Figure 2:**
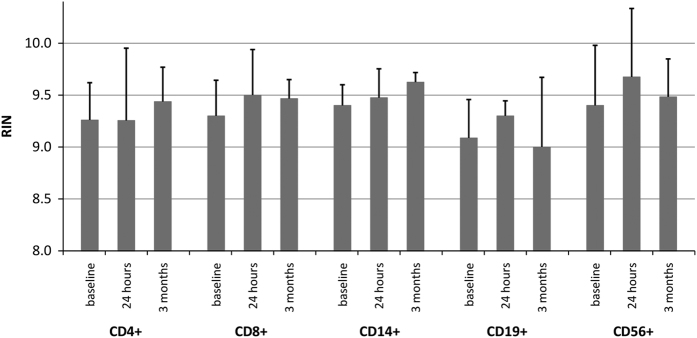
Integrity of the RNA samples. Each bar depicts the arithmetic mean of the RNA integrity numbers (RIN) measured for the 10 whole RNA samples, which were used for the microarray hybridisation per cell population and per time point. The error bars indicate standard deviations. This quality control was carried out using the Agilent Bioanalyzer 2100 and the RNA 6000 Pico LabChip kit (Agilent Technologies, Waldbronn, Germany).

The RNA quality was controlled using standardised operating procedures (SOPs) at the Core Facility for Microarray Analysis of the University of Rostock. The integrity of the RNA samples was checked using an Agilent Bioanalyzer 2100 with the RNA 6000 Pico LabChip kit (both from Agilent Technologies, Waldbronn, Germany). RNA integrity numbers (RIN) were determined using the ratio of 18 S and 28 S ribosomal RNA and other electropherogram characteristics^22^. RINs between 8 and 10 indicate optimal RNA quality for microarray experiments. Our samples grouped around an average of 9.37 (Fig. 2).

One major effect of the treatment with fingolimod is the inhibition of lymphocyte egress from lymphoid tissues towards the peripheral blood^6^. This led especially after 3 months to lower cell counts for certain immune cell subsets^15^. The sample with the lowest RNA yield determined the starting amount for the labelling procedure for all samples from one particular cell population. For CD8+ and CD14+ cells, we used 200 ng, and for CD4+, CD19+ and CD56+ cells, we used 70 ng of total RNA. This amount of starting material was within the limits specified by the manufacturer for the GeneChip WT PLUS Kit (50 to 500 ng).

### Data distribution and hybridisation controls

**Figure 3:**
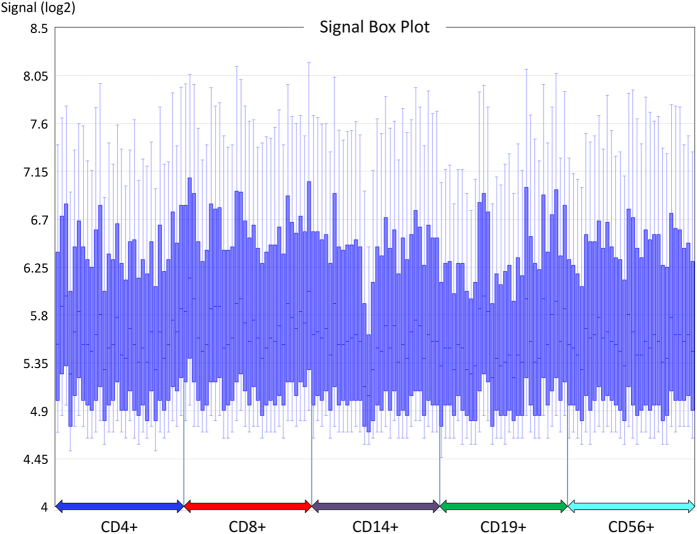
Signal box plot visualising the raw data distribution of the 150 microarrays before normalisation. The blue boxes comprise the probe intensity values (in log2 scale) of 50% of all features (between the upper and lower quartiles). The medians are tagged by horizontal lines. Upper and lower whiskers indicate the 90% and the 10% quantiles, respectively. The data are grouped on the x-axis according to the cell subsets, which were enriched from the blood of patients with multiple sclerosis.

The initial HTA 2.0 microarray data processing was done for each of the 5 cell populations with the Expression Console 1.3.1 software (Affymetrix)^13–15^. We here present quality control assessments that were reconducted for the whole dataset with the latest version of TAC (4.0.1., Applied Biosystems), which also offers more flexible types of comparisons and an advanced RMA method with Signal Space Transformation (SST-RMA). Firstly, a box plot of the raw signal intensity levels of the >6 million probes per array was created, with boxes indicating interquartile ranges and whiskers indicating 10 and 90% percentiles. The resulting graph demonstrates that the data of the 150 CEL files are overall similarly distributed even before normalisation (Fig. 3).

**Figure 4:**
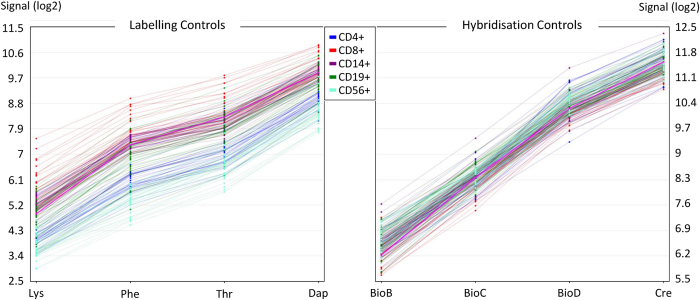
Line graphs of hybridisation controls. Premixed spikes were added to the samples at two time points: before the enzymatic steps (labelling controls) and during the preparation of the hybridisation cocktail (hybridisation controls). They served to control the proper enzymatic conversion of transcripts and the correct conditions during microarray hybridisation, washing, staining and scanning. The labelling controls are sequences derived from *Bacillus subtilis* genes (Lys: AFFX-r2-Bs-lys (1:100,000), Phe: AFFX-r2-Bs-phe (1:50,000), Thr: AFFX-r2-Bs-thr (1:25,000), Dap: AFFX-r2-Bs-dap (1:6,667)). The default hybridisation controls are composed of BioB, BioC, BioD and Cre in staggered concentrations (AFFX-r2-Ec-BioB, AFFX-r2-Ec-BioC, AFFX-r2-Ec-BioD, AFFX-r2-P1-Cre). The two graphs are depicting the respective log2 values after applying SST-RMA. Consistency across all 150 samples is demonstrated by increasing signal values (Lys<Phe<Thr<Dap and BioB<BioC<BioD<Cre). The colour code distinguishes the 5 different cell populations studied.

Secondly, the levels of hybridisation controls were examined. According to our SOPs, we consequently use a spike-in strategy, which is delivered by the GeneChip WT PLUS Reagent Kit (Affymetrix). Staggered amounts of Poly-A RNA spikes are used as labelling controls, which are added to the RNA samples before the first enzymatic reaction. These spikes are derived from 4 *Bacillus subtilis* genes (Dap, Thr, Phe, Lys). Moreover, before the hybridisation, staggered amounts of hybridisation controls are added. These are biotinylated RNA molecules derived from biotin synthesis pathway genes of *Escherichia coli* (BioB, BioC and BioD) and the Cre recombinase of bacteriophage P1. This spike-in approach is conform to the former protocol for Affymetrix 3’ *in vitro* transcription (IVT) microarrays. For the HTA 2.0 dataset, all spike controls where “within bounds” according to the TAC quality control metrics (Fig. 4).

**Figure 5:**
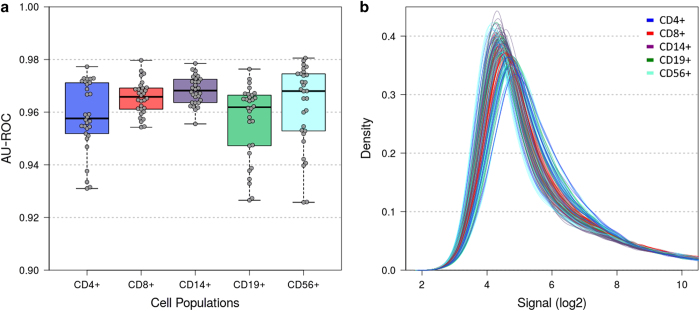
Consistency across samples with respect to data quality and data distribution. (**a**) Analysis of positive and negative control probe sets for quality control. Box plot of parameter values calculated by the area under the curve of the receiver operating characteristic (AU-ROC). The boxes mark the medians and the upper and lower quartiles, and the whiskers extend to the most extreme values. All 150 microarrays showed high AU-ROC values >0.92. (**b**) Density plot displaying the distributions of gene-level probe set signal intensities after SST-RMA normalisation. None of the curves deviated markedly from the others, demonstrating comparable performance.

Finally, we evaluated the signals of positive and negative control probe sets as well as the normalised data of protein-coding and non-coding probe sets. A quality metric was calculated by the area under the receiver operating characteristic curve (AU-ROC) describing the separation of the positive (exon) controls to the negative (intron) controls. An AU-ROC value of 1 reflects perfect separation. The default threshold in the TAC software to flag poor quality arrays is 0.7. For our dataset, the AU-ROC values of all 150 microarrays were >0.92 (Fig. 5). Even higher values (>0.95) resulted for the samples of CD8+ and CD14+ cells, of which 200 ng (instead of 70 ng) of total RNA were used as starting material in the labelling protocol. Additionally, the distribution of the normalised data of all summarised probe sets mapping to genes (n=67,528, excluding 2,995 control probe sets) was inspected for each microarray using a density plot (Fig. 5). The density curves converged in particular for log2 signal intensities >7, revealing good comparability of the expression profiles.

### Gene expression analysis

The expression of selected genes was further inspected for plausibility checks. First, we compared the measured mRNA levels for CD4, CD8A, CD14, MS4A1 (=CD20) and NCAM1 (=CD56) in the processed gene-level data as deposited in the GEO database (Data Citation 1). For the 30 samples of each cell population, the probe set for the respective marker gene always had a relatively high log2 signal intensity of >6.8, which confirmed the successful blood cell separation. Accordingly, the transcriptome data grouped by cell population in the principal component analysis (PCA) as described in our previous publication^15^. The validity of the data has also been confirmed by the Y-chromosomal gene RPS4Y1 (probe set TC0Y000351.hg.1), which had levels <2.32 in all samples from female patients but levels >4.35 in all samples from male patients. When comparing the data of the different time points during therapy, the more than 2-fold downregulation of CCR7 (probe set TC17001466.hg.1) observed in both CD4+ cells and CD8+ cells was also consistent with the mechanism of action of the S1P receptor modulator fingolimod^13,14^.

## Additional information

**How to cite this article**: Koczan, D. *et al.* Microarray data of transcriptome shifts in blood cell subsets during S1P receptor modulator therapy. *Sci. Data* 5:180145 doi: 10.1084/sdata.2018.145 (2018).

**Publisher’s note**: Springer Nature remains neutral with regard to jurisdictional claims in published maps and institutional affiliations.

## Supplementary Material



## Figures and Tables

**Table 1:** SubSeries of the transcriptome profiling data in the GEO repository.

**Cell population/ Probe set definition**	**Storage address for CEL files and CHP files at GEO**
CD4+/Exon level	https://www.ncbi.nlm.nih.gov/geo/query/acc.cgi?acc=GSE73080
CD4+/Gene level	https://www.ncbi.nlm.nih.gov/geo/query/acc.cgi?acc=GSE73079
CD8+/Exon level	https://www.ncbi.nlm.nih.gov/geo/query/acc.cgi?acc=GSE73172
CD8+/Gene level	https://www.ncbi.nlm.nih.gov/geo/query/acc.cgi?acc=GSE73081
CD14+/Exon level	https://www.ncbi.nlm.nih.gov/geo/query/acc.cgi?acc=GSE81603
CD14+/Gene level	https://www.ncbi.nlm.nih.gov/geo/query/acc.cgi?acc=GSE81598
CD19+/Exon level	https://www.ncbi.nlm.nih.gov/geo/query/acc.cgi?acc=GSE81606
CD19+/Gene level	https://www.ncbi.nlm.nih.gov/geo/query/acc.cgi?acc=GSE81604
CD56+/Exon level	https://www.ncbi.nlm.nih.gov/geo/query/acc.cgi?acc=GSE81611
CD56+/Gene level	https://www.ncbi.nlm.nih.gov/geo/query/acc.cgi?acc=GSE81607
